# Steady State Detection of Chemical Reaction Networks Using a Simplified Analytical Method

**DOI:** 10.1371/journal.pone.0010823

**Published:** 2010-06-03

**Authors:** Ivan Martínez-Forero, Antonio Peláez-López, Pablo Villoslada

**Affiliations:** 1 Department of Physics and Applied Mathematics, University of Navarra, Pamplona, Spain; 2 Hospital Clinic, Institute of Biomedical Research August Pi Sunyer (IDIBAPS), Barcelona, Spain; University of Nottingham, United Kingdom

## Abstract

Chemical reaction networks (CRNs) are susceptible to mathematical modelling. The dynamic behavior of CRNs can be investigated by solving the polynomial equations derived from its structure. However, simple CRN give rise to non-linear polynomials that are difficult to resolve. Here we propose a procedure to locate the steady states of CRNs from a formula derived through algebraic geometry methods. We have applied this procedure to define the steady states of a classic CRN that exhibits instability, and to a model of programmed cell death.

## Introduction

**Figure 1:**
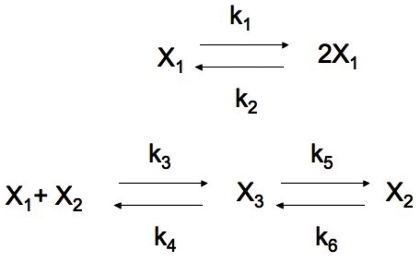
Edelstein chemical reaction network scheme. 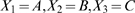
.

Chemical reaction networks (CRNs) display interesting dynamic properties. In order to understand the temporal evolution of chemical species in reactions, CRN are often modeled through systems involving ordinary differential equations. The ODE system derived from a CRN endowed with mass action kinetics is a polynomial system of several variables. The qualitative behavior of CRNs can be outlined if we can find the stationary solutions to such ODEs. However, most of the time these polynomials are non-linear, making them hard to resolve. In an attempt to circumvent this problem, well known theories have attempted to elucidate the qualititave dynamics of CRNs using methods applied to CRN structure alone (i.e Feinberg's Chemical Reaction Network Theory and Clarke's Stoichiometric Network Analysis). Here, we present a theory necessary to understand the dynamic properties of CRNs and accordingly, throughout the text we will follow a classic biochemical reaction network. In 1970, Edelstein proposed a reaction scheme that has multiple steady states and a hysteresis loop [1]. The structure of the model is displayed in Figure 1, whereb the network is composed of three species (A,B and C) and six reactions. The chemical mechanism represented is that of species A autocatalytic production and posterior enzymatic degradation. During the explanation we will assume that chemical reactions occur in a well stirred chemical reactor at constant temperature.

## Results

### Definitions

A reaction network is composed of three sets [2]:

- Species: the chemical components of the network; 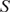

- Complexes: the formal combinations of species that appear before and after of reaction arrows; 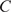

- Reactions: specify how complexes are joined by arrows. 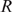

In the Edelstein model 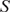 = {A,B,C}, 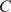 = {A, 2A, A+B, C,B} and 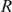 = {A
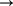
2A,2A
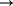
A,A+B
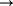
C, C
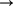
A+B, C
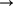
B,B
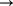
C}, the number of species 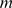, the number of complexes 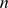 and the number of reactions 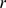 in the example being dealt with is 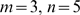 and 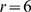.

Each chemical entity is associated with a continous variable representing its concentration (measured in moles per litre, 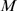, or in another appropriate unit). Only non-negative concentrations are biologically realistic and we will use 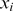 to identify the concentrations of different species. In this way A = 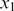, B = 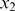 and C = 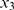. Complexes are denoted 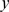 and they may be reactant complexes 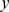 or product complexes 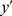. Reactions are represented as 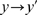. A complex vector contains the stoichiometric coefficient of species 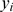 in complex 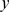. In open systems we refer to a special complex, known as the zero complex **0**, for which all entries are 0 and that has as many entries as the number of species in the system under study. As an example, the complex vector for complex 2A is 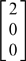.

The complex matrix 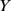 is a 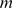
×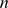 matrix that contains the complex vectors as columns. A reaction vector is the vector resulting from the subtraction of the reactant complex from the product complex, 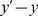. For the reaction A+B
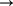
C the reaction vector is 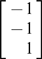.

The stoichiometric matrix, N, is 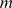
×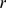 in size and its columns represent the reaction vectors of the chemical network. For the Edelstein model we obtain
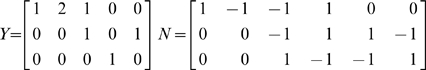
(1)In general, chemical networks have conserved relationships that can be identified by calculating the left null space of N. If 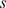 is the rank of N, there are 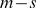 conserved relationships. Therefore, in our working example 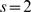, there is a relationship of conservation 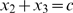. The conservation relationship gives rise to stoichiometric compatibility classes that have important consequences in the study of CRN equilibrium solutions.

The kinetics for a reaction network {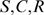} involve a function that describes the rate at which the chemical species interact to form products. The most common kinetics implemented so far are mass action kinetics (MA). In MA, the rate of the reaction is proportional to the product of the concentration of the reactant species and a kinetic constant 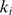. The general form of MA is
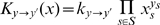
(2)where 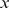 is the concentration vector. In these MA, the reaction parameters are positive, and the are estimated using chemical principles or they are deduced from experiments. It is noteworthy that accurate values for such parameters are not often known for complex chemical networks. The reaction rates form a vector 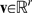, which in the Edelstein case is 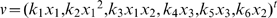


The matrix N can be viewed as the multiplication of two matrices 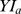 where 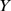 is the complex matrix and 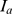 is an 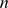
×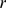 incidence matrix [2]. Each column of 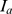 represents a reaction and has an entry −1 for the reactant complex and 1 for the product complex. Likewise, the reaction vector v is the product of 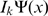. 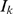 is a 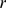
×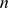 matrix containing the rows of the kinetic constants for each reaction, 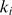 for reactants. 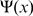 is a monomial vector for the species participating in each complex [3]. For the Edelstein example
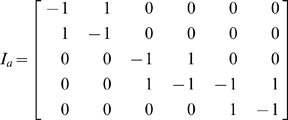
(3)

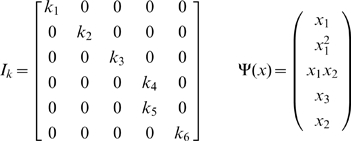
(4)The ODE system for a chemical network is of the form
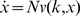
(5)where N is the stoichiometric matrix and 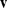 is the reaction vector. Using the decomposition previously explained, the ODE system is also presented in the following form
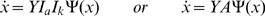
(6)According to these considerations, the differential equations for the Edelstein network are
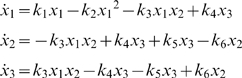
(7)As a final definition is needed. The stoichiometric subspace for a reaction network is the linear subspace defined by
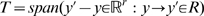
(8)In our example, the stoichiometric subspace is generated by the reaction vectors {C-B,A}. The significance of T is that the concentration of each chemical is constrained to evolve in an defined subspace, which is a parallel translation of T. Stoichiometric compatibility classes are parallel translates of the stoichiometric subspace.

### Equilibrium solutions

In the previous section we explained a framework for CRNs. Starting from the structure of chemical reactions, it is possible to derive an ODE system for the dynamical study of CRN in a unique and orderly way. Differential equations obtained from a CRN are tied to the network structure. Thus, from this point on if we know the reaction parameters (with appropriate units) and initial conditions, we can commence a numerical analysis of the systems to determine how the species' concentrations change over time. If parameters are difficult to obtain, it would be desirable to gain some insight into the dynamic capacities of the CRN using reaction structure alone. This approach has been promoted and called “complex biology with no parameters” [4]. In order to understand a CRN we would like to solve the vectorial equation 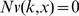 to determine the stationary states where the system converges. Thus, we are faced with the need to resolve several variables of a non-linear polynomial system. Two general theories have adressed this issue : Feinberg's Chemical Reaction Network Theory (CRNT) and Clarke's Stoichiometric Network Analysis (SNA) [2] [5]. SNA and CRNT are methodologies to study the qualitative dynamic behavior of chemical networks [6]. CRNT has received special attention in recent years as it is a reliable method to rule out hypotheses about the mechanism of a particular CRN [7] [8] [9] [10] [11] [12]. If other tools (i.e. SNA and CRNT) can identify the possibility of a certain dynamic behavior, the method we explain in the following sections provides an essential tool to determine where this behavior might occur.

### Region of multistationarity

**Figure 2:**
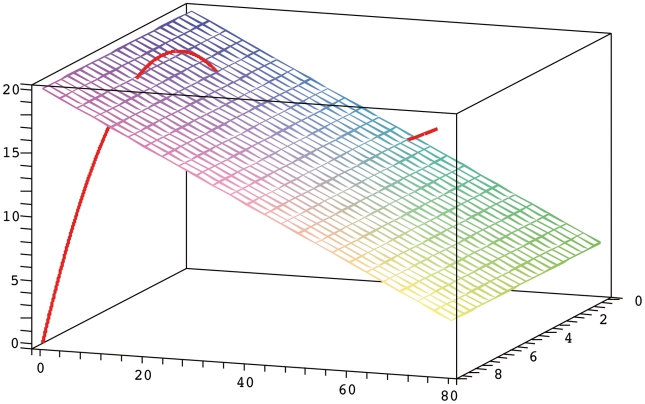
Number of equilibrium solutions for the Edelstein system by changing the value of conservation relation. Parameter values are 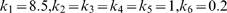.

In many cases SNA and CRNT can decide whether or not a specific CRN is capable of displaying multistationarity. However, it is still necessary to locate the region where this property might appear, and algebraic geometry methods are a natural choice to address that need. To ilustrate how to use algebraic geometry to reveal the site of multistationarity, we will continue dissecting the Edelstein network. As already mentioned, this network displays multistationarity for certain values of the reaction parameters. Figure 2 shows how according to the different locations of the equilibrium curve and the stoichiometric compatibility class intersections, there may be one, two or even three steady states. In order to identify the exact points of intersection we followed the procedure below:

1. Reduce N to its row reduced echelon form, RD;
2. Identify stoichiometric compatibility classes;
3. Based on the RD construct, new equations are derived by multiplying RD by the vector of reaction rates **v**(k,x);
4. Add the equation representing stoichiometric compatibility classes to the previous system (conservation relationships). We will call this new equation system AD;
5. Calculate the Gröbner basis of AD using an elimination order (i.e. lexicographic order);
6. Normally, this basis will produce a set of polynomials arranged in echelon form.

**Figure 3:**
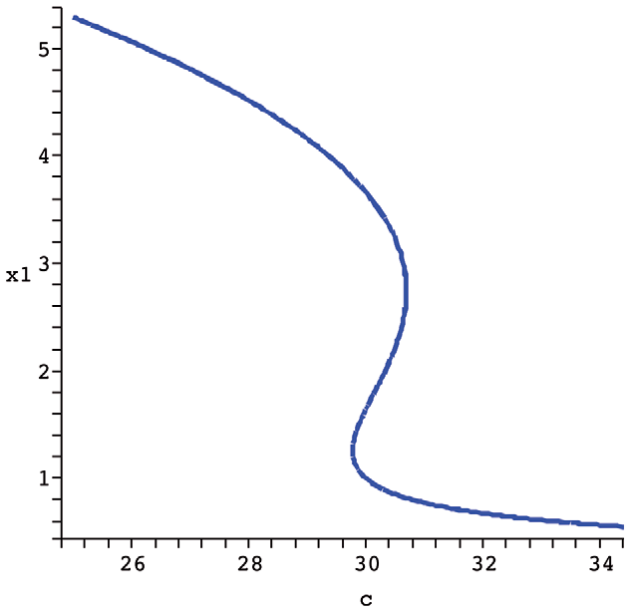
Bifurcation diagram 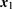 vs 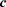. Parameter values are the same as in Figure 2.

The procedure for the Edelstein system yielded the following result:
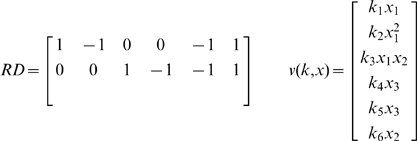


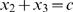
The new system AD is
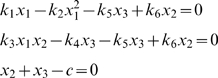
(9)Now we can calculate the Gröbner basis for the new system. 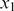 represents the chemical product in the Edelstein network and thus, it is of interest to represent equilibrium solutions of 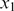 in terms of the different 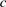 values. The MAPLE command to obtain the basis is gbasis([f,g,h],plex(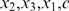)), where f,g,h are each of the elements in the polynomial system AD. The complete basis is a huge polynomial system and therefore, we have not reproduced it here. Using the parameters described previously [2], the first element of the basis is 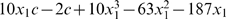. A diagram of the solution for 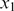 in terms of 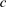 appears in Figure 3, based on the formula obtained analytically 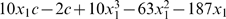. It is evident that multistationarity is only possible for a small range of 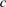. Algebraic geometry methods allowed us to identify in which interval of the stoichiometric compatibility class multiple steady states exist. When correctly applied, the method developed in this section is able to identify the region of multistationarity. We would like to highlight that the procedure remains silent in terms of the local stability of the computed steady states. In order to determine the stability, it is neccesary to calculate the eigenvalues of the Jacobian matrix evaluated at the specified steady state. Below we have used the method described to analyse a mathematical model of apoptosis.

### Aplication of Gröbner basis for the study of apoptosis

Apoptosis is an essential process to maintain homeostasis in organisms. Abnormalities in the control of apoptosis can promote the development of autoimmune diseases, neurodegenerative diseases or cancer. Thus, understanding the apoptosis machinery is of considerable biological and medical interest. Apoptosis is a suitable system for mathematical modeling. First, it is complex, by which we mean that its collective properties cannot be explained from the study of each component in isolation. Second, it displays a qualitative property (bistability) useful to model validation. Third, the central mechanism of apoptosis is well known and the parameters for ODE simulation are available in the literature. In this regard various attempts to model apoptosis have been published [13] [14] [15].

**Figure 4:**
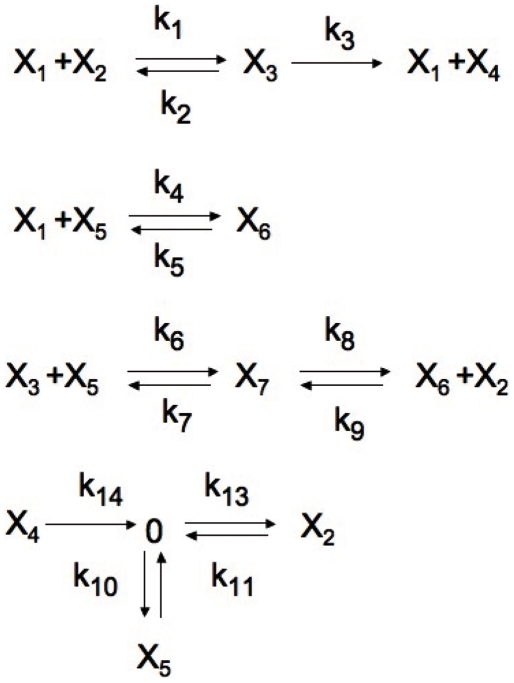
A new model for receptor induced apoptosis.

Based on our current knowledge of how apoptosis is regulated, we describe here a new model for receptor induced cell death. The CRN represents Caspase 8 dependent activation of Caspase 3 and inhibition of apoptosis mediated by BAR (Bifunctional Apoptosis Regulator) [16] [17]. Figure 4 represents a diagram of the proposed model. The model has seven species and fourteen reactions. The species are:

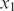
 = Activated Caspase 8 (C8*)

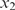
 = Caspase 3 (C3)

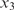
 = The C8*C3 Complex

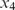
 = Activated Caspase 3 (C3*)

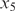
 = Inhibitor of apoptosis (BAR)

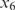
 = The C8*BAR Complex

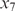
 = The C8*C3BAR Complex

The reaction rates conform to the vector 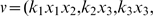 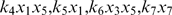 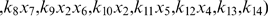


The ODE system for this network is
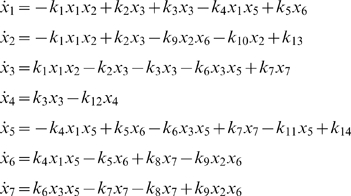
(10)There is a conserved relation for total C8* that is represented by 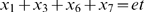. We would like to know if our model displays bistability as required. In order to verify this issue, the parameters reported for some of the reactions in the apoptosis network were used in a numerical analysis [18] using the procedure explained above. The complete derivation can be followed with a MAPLE file available upon request.

**Figure 5:**
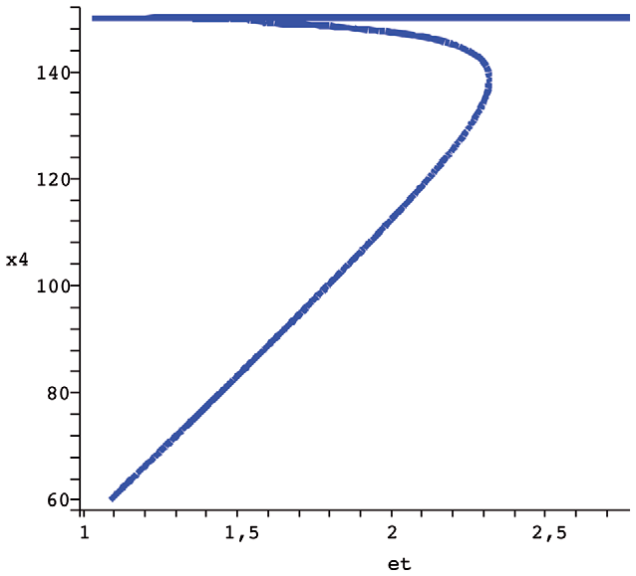
Bifurcation diagram 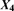 vs et. The parameters used are 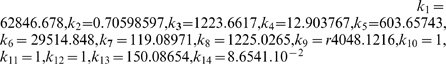.

**Figure 6:**
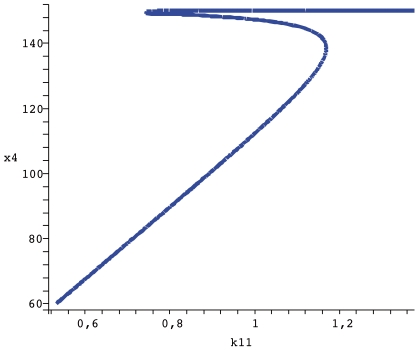
Bifurcation diagram 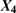 vs 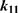. The remaining parameters are the same as in Figure 5 and 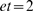.

If we use the 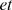 conservation relation as a parameter of bifurcation, it is possible in the model now proposed to admit three stady states in a range of total C8*, two stable and one unstable. In Figure 5 the bifurcation diagram for the apoptosis system obtained through the analysis described above is shown. Slight changes in turnover rates influence the dynamic behavior of CRNs and now, we wish to carry out a similar procedure for the apoptosis model proposed. The parameter 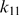 controls the degradation of BAR (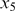), an inhibitor of caspase activation. The bifurcation diagram in Figure 6 shows how even for a region that is supposed to display multistationarity (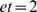 according to Figure 5) mild variations in 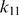 allows the system to commute between low and high levels of executioner caspase 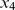 (cleaved caspase 3). This is interesting because if a pharmacological perturbation does not interfere with the total amount of the initiator caspase (Total C8 = 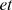), the system can be controled with drugs that promote or inhibit 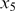 degradation. The clinical implication is if the physician wants to promote apoptosis (i.e. in cancer cells), a temporary increase shoudl be induced 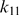, whereas to inhibit apoptosis he should just prescribe 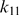 a transient reduction.

## Discussion

In this work we propose a new method to analyse CRNs based on algebraic geometry and we have applied this method to two well known biochemical examples. The method is useful to find the locus of multistationarity in CRNs that display this property in a fully analytical way. Very recently, various groups provided key insights into the application of algebraic geometry to study CRNs [19]–[20], yet we believe that the procedure developed here stands out due to its simplicity and resolving power. However, the high computational cost underlying the calculation of Gröbner basis is a limitation when using algebraic geometry methods. This problem can be overcome by dividing the CRN into subnetworks, resolving each subnetwork and then applying the results to the overall CRN [21]. We evaluated a model for the mechanisms of apoptosis and instead of a simulation approach, we used an analysis based only on the structure of the reaction network. This parameter-free approximation has gained considerable attention in the field of systems biology [21] [4]. In particular the relation between the structure of the network and the qualitative properties inherent to the system (like bistability) is of great importance due to the difficulty in identifying reliable reaction parameters [22].

Chemical reactions are usually modeled by lumping together reactions and ignoring the behavior of intermediary products. This can lead to different dynamic properties if one compares the behavior of complete mechanisms and their lumped counterpart. For example using CRNT, it was recently shown that a simple model of enzyme catalysis that exhibits multistationarity lost this property by neglecting enzyme-substrate intermediates [23]. Representing chemical reactions as accurately as possible is essential when developing appropriate mathematical models of cellular processes.

In summary our results illustrate the power of algebraic geometry methods to evaluate the dynamic capabilities of a chemical reaction network.

## Materials and Methods

The ODE system derived from a CRN endowed with mass action kinetics is a polynomial system. Most of the time these polynomials are non-linear, making it difficult to calculate the steady states. During the last few years, there has been growing interest in applying algebraic geometry methods to the study of CRNs in equilibrium [24] [25] and in particular, Karin Gaterman's work trying to link CRNT and SNA through toric ideals deserves a special mention [3]. In order to exploit the capabilities of algebraic geometry, we will briefly review the main concepts required to deal with CRNs, while referring the interested reader to an excellent treatise on this topic. [26]

We will first broadly define what is a ring. A ring is a set where the addition, subtraction and multiplication operations can be defined with the usual properties (commutative, distributive, etc). If the non-null elements have an inverse, the ring is now a field. In this context the set of real numbers 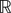 is a field while the integers 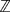 are a ring. A monomial in 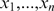 is a product of the form 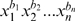, where 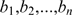 are non-negative integers. For example, 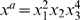 is a monomial and 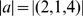 is the grade of the monomial. A polynomial is a combination of monomials that can be represented in the following form 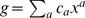 where 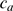 are coefficients. Taking a coefficient field 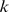, 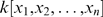 denotes the ring of all polynomials in 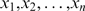 with coefficients in 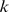. An ideal 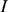 is a subset of 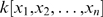 if itsatisfies the following conditions [26]:

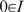
;
- If 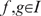 then 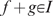;
- If 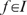 and 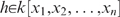 then 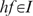.

This definition is used to understand the Hilbert Basis Theorem that states that every ideal in 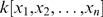 is finitely generated. A set of generators of an ideal is called a basis. That is, there exists 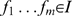 such that 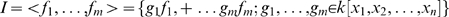. A variety is the set of solutions of a polynomial system. We can consider the system 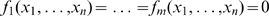 and the variety 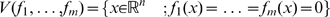. The ideal 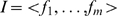 contains infinite polynomials, but 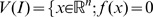 for all 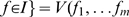). For this reason, to find the solutions of the system we are interested in, an adequate basis of 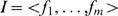 must be obtained. If we are willing to solve the equation 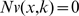, we would like to get a basis that permits us to eliminate some variables and to back-substitute to obtain the value of the remainder variables. One type of generator or basis that permits elimination theory for an ideal to be applied is the Gröbner basis with lexicographic order. The definitions of the Gröbner basis and lexicographic order are found below, but first it is important to define what an order means. As stated before a polynomial is a combination of monomials. An order is a procedure to exactly rearrange the terms of a polynomial in an ascending or descending manner. Several monomial orderings have been described including lexicographic (lex), graded lexicographic (grlex) and graded reverse lexicographic orders (grevlex). A Gröbner basis for an ideal 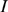 is that in which the polynomial remainder with respect to the basis determines the membership of 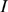. It is considered a basic result that a Gröbner basis always exist for any ideal and any monomial order, but the result may differ according to the monomial order of choice. General mathematical software, such as MAPLE and Mathematica, have implementations of algorithms to calculate the Gröbner basis. The Gröbner basis obtained in this work were determined using the Groebner package in MAPLE. The computational cost of calculating a Gröbner basis is extensive and some problems are almost never solved in a realistic timescale, even if theoretically it is always possible to obtain a Gröbner basis for an ideal. The main use of this type of calculations is to find the solutions of polynomial systems. The idea behind applying algebraic geometry to CRNs is that the ODE system derived from a CRN endowed with mass action kinetics is a polynomial set conformed by monomials representing the rates of production and elimination of chemical species. If other tools (i.e. SNA and CRNT) enable the possibility of a certain dynamic behavior to be identified, algebraic geometry is an essential tool to find where this behavior can appear.
